# Fluoroquinolone resistance and mutational profile of *gyrA* in pulmonary MDR tuberculosis patients

**DOI:** 10.1186/s12890-020-1172-4

**Published:** 2020-05-11

**Authors:** Saba Kabir, Zarfishan Tahir, Nadia Mukhtar, Muhammad Sohail, Muhammad Saqalein, Abdul Rehman

**Affiliations:** 1 Department of Microbiology and Molecular Genetics (MMG), University of the Punjab, New Campus Lahore, Lahore, 54590 Pakistan; 2 Institute of Public Health, Lahore, Pakistan; 3 University of Veterinary and Animal Sciences, Lahore, Pakistan; 4 Government College University, Faisalabad, Pakistan

**Keywords:** *Mycobacterium tuberculosis*, MDR and pre-XDR TB, Fluoroquinolone resistance, MTBDR*sl* assay

## Abstract

**Background:**

Fluoroquinolones (FQs) are potential drugs that inhibit DNA synthesis and are used in the treatment of multidrug-resistant tuberculosis (TB) and short-term anti-TB regimens. In recent years, a high proportion of FQ resistance has been observed in *Mycobacterium tuberculosis* isolates. The development of FQ resistance in multidrug-resistant TB negatively impacts patient treatment outcome and is a serious threat to control of TB.

**Methods:**

The study included a total of 562 samples from patients with pulmonary TB that had been on anti-tuberculosis therapy. MTBDR*sl* assays were performed for the molecular detection of mutations. Sequence analysis was performed for the characterization and mutational profiling of FQ-resistant isolates.

**Results:**

FQ resistance was observed in 104 samples (18.5%), most of which were previously treated and treatment failure cases. A total of 102 isolates had mutations in DNA gyrase subunit A (*gyrA*), while mutations in *gyrB* were observed in only two isolates. Mutational analysis revealed that the mutations mostly alter codons 94 (replacing aspartic acid with glycine, D94G) and 90 (replacing alanine with valine, A90V). In MDR and treatment failure cases, resistance to FQs was most commonly associated with the D94G mutation. In contract, a high proportion of A90V mutations were observed in isolates that were newly diagnosed.

**Conclusion:**

The findings suggest that genotypic assays for FQ resistance should be carried out at the time of initial diagnosis, before starting treatment, in order to rule out mutations that impact the potential use of FQs in treatment and to control drug resistance.

## Background

Pakistan is among the thirty countries with high tuberculosis (TB) burden in which the complete elimination of TB is, unfortunately, a distant reality. Worldwide, TB is ranked as the ninth leading cause of mortality, with about one third of the world’s population having latent TB infection [1]. According to the 2018 annual report of the World Health Organization (WHO), the incidence of TB in Pakistan is 267/100,000 population and the mortality rate is 27/100,000 (excluding cases with HIV) [2]. There is a significant proportion of the population in which TB remains undiagnosed and untreated.

Fluoroquinolones (FQs) have long been used as anti-tuberculosis drugs, and their wide spread use has led to the development of resistance in clinical isolates of *Mycobacterium tuberculosis* (MTB). Phenotypic resistance of TB to FQ is associated with mutation in the quinolone resistance-determining region (QRDR) of DNA subunits A (*gyrA*) and B (*gyrB*), which encode a type II DNA topoisomerase. Mutations in subunit A confer high-level resistance, whereas those in subunit B confer low-level resistance [3]. During treatment of TB, multidrug-resistant (MDR) patients can develop resistance against fluoroquinolones. The development of such resistance is a risk factor can aid in the transition of these patients from MDR to pre-extensively drug resistant (pre-XDR) TB, and they can become extensively drug resistant with further resistance to at least one injectable second -line drug [4, 5].

In *gyrA*, the most common mutations appear in codons 88–94 of the QRDR, particularly in codons 88, 90, 91, and 94. In *gyrB*, FQ resistance is most commonly associated with mutations in codons 500 and 538 [6]. However, geographic differences in the frequency of *gyrA* mutations are known to exist. Understanding the frequency and geographic distribution of FQ resistance mutations is important in order to maximize the sensitivity and specificity of treatment.

The emergence of drug resistance and persistence of infection is a serious threat to the control of TB [7]. A high incidence of resistance severely limits treatment options and requires the use of more toxic and costly treatment regimens [8]. The present study was conducted to detect the mutational profile of fluoroquinolone resistance in order to help in determining the potential utility and selection of adequate drug regimens.

## Methods

### Sample collection

*Mycobacterium tuberculosis* isolates were procured from patients diagnosed with pulmonary TB. The samples were collected from sites involved in programmatic management of drug resistant tuberculosis (PMDT) within seven different districts (Lahore, Faisalabad, Gujranwala, Sahiwal, Sargodha, Sialkot, and Bahawalpur) from period of May 2018 to March 2019. A total of 562 suspected MDR-TB cases were included in the study. GeneExpert and MTBDR plus assays were performed to test isolate susceptibility against first-line anti-TB drugs. Histories of anti-tuberculosis treatment were obtained from patients, which included newly diagnosed cases and previously treated cases (treatment failure and treatment default).

This study was approved by the Research Ethics and Biosafety Committee (No.D/650/MMG) of the Department of Microbiology and Molecular Genetics, University of the Punjab, Lahore, Pakistan.

### Sample processing

Initially, sputum samples were decontaminated using the standard N-acetyl-L-cysteine sodium hydroxide (NALC-NaOH) method [9], and smear-positive samples were directly processed for DNA extraction. Smear-negative samples were primarily cultured on MGIT Bactec 960® medium. DNA was extracted using Genolyse version 1.0 kits (Hain Lifescience, Germany). After extraction, the supernatant was collected, transferred into a fresh tube, and stored at -20 °C for further processing.

### Molecular detection of FQ resistance

Phenotypic resistance to second-line drugs including fluoroquinolones was determined using GenoType MTBDR*sl* version 2.0 kits. The procedure for molecular detection with GenoType MTBDR*sl* includes consists of three steps: DNA extraction, amplification with biotinylated primers, and reverse hybridization. A test was considered valid when all control bands appeared correctly.

### PCR and sequencing

Primers were designed against the QRDR region of *gyrA*: forward primer 5-GATGCAGCGCAGCTACATCGAC-3 and reverse primer 5-GATGCAGCGCAGCTACATCGAC-3. Thermocycler parameters for the amplification reaction were: 95 °C for 5 min followed by 40 cycles of 95 °C for 30 s, 61 °C for 45 s, and 72 °C for 50 s, with a final elongation of 10 min at 72 °C. The PCR products were purified using a Qiagen PCR purification kit and eluted in TE buffer. The sequencing of isolates was performed by 1st BASE, Malaysia.

## Results

GeneExpert assays were performed for the confirmation of MDR-TB. Susceptibility to first-line drugs was determined against rifampicin (RIF) and isoniazid (INH). Of the 562 samples tested, 430 (76%) were MDR (resistant to both isoniazid and rifampicin), 91 (16%) were mono-resistant [57 (10%) to RIF and 34 (6%) to INH)], and 41(7%) were susceptible to these two drugs.

In terms of treatment history, of the 562 cases, 313 (56%) were newly diagnosed, 97 (17%) were cases of treatment failure (completed treatment but still positive for MTB), 59 (10%) were cases of treatment default (previously took anti-tuberculosis therapy for at least for 1 month, but did not complete treatment), and 93 (17%) had unknown treatment history.

### Detection of FQ resistance

Resistance to FQ and mutational profiling was determined for the DNA gyrase genes *gyrA* and *gyrB*. GenoType MTBDR*sl* assays were performed to determine resistance to second-line drugs.

Resistance was interpreted according to the presence and absence of wild-type and mutant probes. The presence of all wild-types was interpreted to indicate no detectable mutation. The absence of any wild-type probe indicated that probe could not bind the respective amplicon and was considered a detectable mutation.

A total of 104 samples (18.5%) were found to be resistant to FQs. Among these isolates, eight (8%) were rifampicin-sensitive, eight (8%) were mono-resistant (resistant to rifampicin and sensitive to isoniazid), and 88 (85%) were MDR. Most of the patients had been treated previously and appeared with failure of category 1; some were on category 4 treatment. In total, 102/104 of resistant samples showed resistance mutations in*gyrA*, while 2/104 cases had a mutation in *gyrB* (both E540V).

### Mutational profiling of *gyrA*

The mutation probes in the MTBDR*sl* assays detect some of the most common resistance-mediating mutations. Table 1 shows all types of mutations in the *gyrA* gene and the corresponding patterns of wild-type and mutant bands. The most common failure was of the *gyrA* WT3 band and development of MUT3C the most common mutation. Mutational analysis revealed that these mutations mostly alter the protein at codons 94 and 90, respectively replacing aspartic acid with glycine and alanine with valine. The aspartic acid at codon 94 was also replaced with alanine, asparagine, and tyrosine in other types of mutations, and mutation at codon 91 where serine was replaced with proline was also observed.

**Table 1:** The frequency and mutations confer resistance to fluoroquinolones

gyrA Mutation probe	Missing wild type probe	Phenotypic Susceptibility	Mutation	Frequency (***n*** = 102)
gyr A MUT1	gyr A WT2	Resistant	A90V	25
gyr A MUT2	gyr A WT2 gyr A WT3	Resistant	S91P	7
gyr A MUT3A	gyr A WT3	Resistant	D94A	7
gyr A MUT3B	gyr A WT3	Resistant	D94N/Y	9
gyr A MUT3C	gyr A WT3	Resistant	D94G	45
None	any one wild type probe	Resistant	A90V D94A D94G	9

**Fig. 1:**
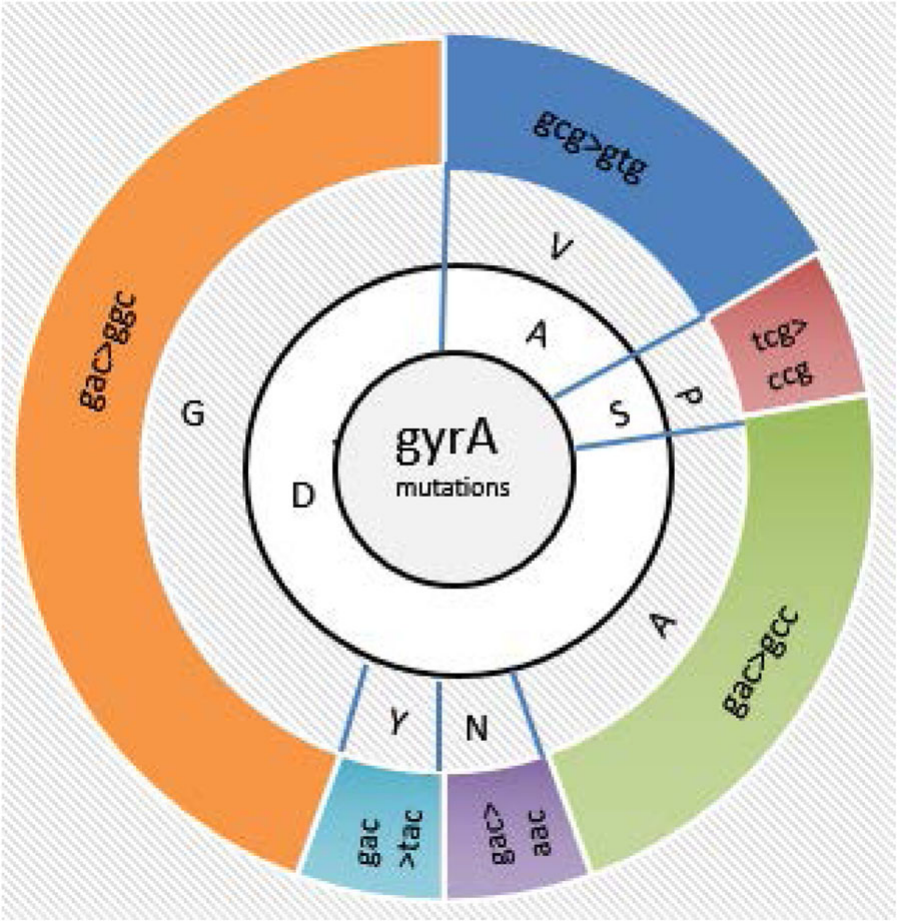
The frequency of amino acid and nucleotide change in fluoroquinolone resistant isolates. In general, amino acid change found in three amino acids: alanine (A into V), serine (S into P), and aspartic acid (D either into A, N, Y or G) and the corresponding nucleoide change to these amino acids is also represented

As different hybridization patterns of the hot spot regions of *gyrA* were observed 19 isolates representing all possible patterns were selected for further mutational analysis, with H37RV (AGH06049.1) used as a reference strain. These mutations were found to result in amino acid changes. Specifically, we identified four isolates which the amino acid changes varied from the most mutation reported by Line probe assay (LPA) testing. One isolate substituted asp→ala, two isolates had ala→val, and one had a thr → ala mutation in addition to ser/ala→X (an undetermined mutation) (Fig. 1).

## Discussion

Fluoroquinolones have long been widely used to treat several infectious diseases and in certain regions are easily accessible even without prescription. Such misuse of FQs has highly contributed to their efficacy in the treatment of TB but also the emergence of FQ-resistance [10].

The current cross-sectional study included presumptive multi-drug-resistant isolates of MTB. High proportions of resistance to rifampicin (RIF) and isoniazid (INH) were observed. Of all samples tested, resistance to first-line drugs (INH and RIF) was identified in 92% (521/562, i.e. 430 MDR and 91 mono-resistant). Among FQ-resistant isolates, eight were RIF-sensitive, eight were mono-resistant to RIF, and 88 were MDR. Isolates having resistance against rifampicin and isoniazid are termed MDR-TB, and if they also develop additional resistance against FQs, are then known as pre-XDR TB [11].

MTB develops resistance against FQs mainly by developing mutations in the drug-targeted proteins. The detection of gyrase mutations in particular can help in predicting the presence and level of FQ resistance [12]. GenoType MTBDR*sl* assay can detect mutations in the conserved QRDR region of gyrase genes (*gyrA* and *gyrB*) which changes the structure of the drug binding pocket (QBP) and results in cross resistance to all FQs. GenoType MTBDR*sl* assays were used to determine the frequency of FQ resistance in our isolates. A total of 104/562 isolates (18.5%) were found to be resistant to FQs. A high prevalence of FQ resistance was also reported in other provinces of Pakistan [10, 13] and in the neighboring countries of India [14, 15], China [16, 17], and Bangladesh [18, 19].

Short treatment regimens are used to reduce emergence of antimicrobial resistance in MTB. According to the 2019 national guidelines for the control of TB in Pakistan (adopted by the WHO), the anti-TB short regimens for drug-susceptible cases include third- or fourth -generation fluoroquinolones (levofloxacin or moxifloxacin, respectively) for 4 months. These drugs are also given for isoniazid-resistant and previously-treated cases in the initial phase of therapy (2 months). The high proportion of FQ resistance indicates an ineligibility of patients in this population for shorter regimens.

**Fig. 2:**
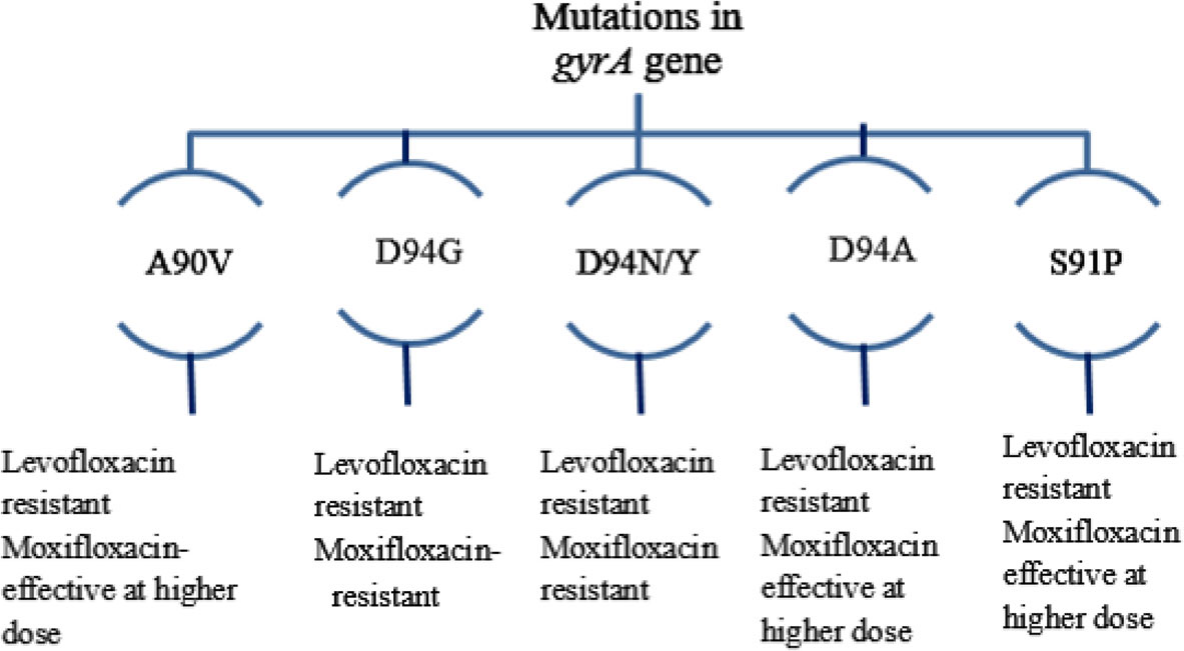
The association of *gyrA* mutation with levofloxacin and moxifloxacin resistance

Among participants in this study, the frequency of *gyrA* mutations was much higher than that for *gyrB*. Hybridization pattern analysis revealed that most isolates had a mutation in the *gyrA* gene locus, with substitutions at amino acid 94 (D94G) and 90 (A90V) being more prevalent. Both of these mutations are associated with a high level of resistance to fluoroquinolone antibiotics (Fig. 2). The A90V mutation confers resistance against levofloxacin, but a higher generation of FQ (i.e. moxifloxacin) can be used at a higher dose. However, if the D94G mutation is present, both levofloxacin and moxifloxacin are ineffective [20]. Additional mutations found in our isolates were S91P, D94A, and D94N/Y. Isolates with the first two mutations could be susceptible to moxifloxacin at higher doses but resistant to levofloxacin. However, D94N/Y confers resistance against both levofloxacin and moxifloxacin. These findings correlate with most of the previous studies [6, 21, 22].

This study assessed patient characteristics and type of mutation in two ways. The first approach was to determine the frequency of particular mutations when categorized according to their resistance to first-line drugs, and the second when categorized according to treatment history. In MDR and XRD cases, FQ resistance was most commonly associated with the D94G mutation. This mutation shows high-level resistance to all fluoroquinolones, even the fourth-generation moxifloxacin. Isolates with INH monoresistance exhibited the S91P and D94N/Y mutations, with D94N/Y also being observed in XRD. Newly-diagnosed TB isolates with FQ resistance showed a high proportion of A90V mutations, for which moxifloxacin still remains the drug of choice at higher doses. In contrast, treatment failure and relapse cases commonly exhibited the D94G mutation.

Since GenoType MTBDR*sl* assay targets only a small region of a gene and supports a limited number of well-known mutations, interpretation in terms of the cross-resistance to FQs that occurs due to particular *gyrA* mutations is sometimes indistinct. Accordingly, sequence analysis was performed to understand resistance at the genotypic level [23]. Some isolates (4/102) showed co-existence of mutations in a hybridization pattern. The combinations identified were: D94Awith D94H, S91P with D94G, D94G with D94N/Y, and A90V with D94G. Co-existence of mutations was also observed in sequence analysis of *gyrA*, with the S91T mutation being detected in 95% isolates. However, this mutation is not related to fluoroquinolone resistance, other studies have determined it could be present even in sensitive isolates [24, 25]. All of our mutation results are in line with a study conducted in Pakistan that detected mutations in extensively drug-resistant strains [26].

Interestingly, we found some other hot-spot mutations during hybridization pattern analysis, wherein all wild-type probes were present and co-existed with a mutant probe. In these two isolates, when MUT3C and MUT2 appeared in the presence of the associated wild-type probe, a substitution of Ala with Val was observed and when the MUT2 probe was present but WT3 absent, a mutation of Asp to Ala occurred. Four isolates exhibited our most common pattern, i.e. absence of WT3 and presence of the corresponding mutant probe MUT3C, but one isolate exhibited a mutation of Thr into Ala combined with G/M/R into an undetermined amino acid (X). However, for most isolates that deviated from the pattern of well-known mutations, the affected amino acids mostly occurred at typical sites, i.e. codons 94 and 91. Overall, these findings suggest that mutation patterns can differ according and this can be revealed in the hybridization patterns of wild-type and mutant probes.

The results of this study present a burden of fluoroquinolone resistance in MDR-TB patients regardless of FQ antibiotic therapy. However, further understanding of the relevance of genotypic and phenotypic resistance is important for the accurate prediction of FQ resistance. Although we found genotypes consistent with FQ resistance in susceptible isolates, these results do not reflect the true prevalence of resistance in these patients. The resistance might develop due to the prior use of FQ antibiotics, which information was not included in the study.

## Conclusions

The emergence of fluoroquinlone resistance in clinical isolates is alarming. We found a high proportion of MDR cases to have FQ resistant genotypes, even in mono-resistant and drug-sensitive isolates. The groups most likely to develop resistance are previously-treated and treatment-failure cases. Our findings suggest that the implementation of FQs in these patients should be carefully administered and that genotypic studies should be carried out, preferably at the time of initial diagnosis, to rule out all types of resistance-promoting mutations in order to ensure effective treatment and particularly to control resistance.

## Data Availability

The datasets generated and/ or analyzed during the current study are part of PhD thesis of the first author and not publicly available. The datasets are available from the corresponding author on reasonable request.

## Abbreviations

FQ: Fluoroquinolone
FQ-R: Fluoroquinolone Resistance
LPA: Line Probe Assay
MDR: Multidrug Resistant
MGIT: Mycobacteria Growth Indicator Tube
Mtb: *Mycobacterium tuberculosis*
MUT: Mutant
NALC-NaOH: N-acetyl-L-Cysteine Sodium Hydroxide
QRDR: Quinolone Resistance Determining Region
TB: Tuberculosis
WT: Wild Type
XDR: Extensively Drug Resistant
